# Atraumatic Medullary Osteonecrosis of the Tibia and Femur Treated With Intraosseous Orthobiologics

**DOI:** 10.7759/cureus.16677

**Published:** 2021-07-27

**Authors:** Adam Street, Mairin A Jerome, Christopher J Williams

**Affiliations:** 1 Department of Orthopedics and Sports Medicine, Emory University, Atlanta, USA; 2 Interventional Orthopedics and Regenerative Medicine, Interventional Orthopedics of Atlanta, Atlanta, USA; 3 Physical Medicine and Rehabilitation, Regenerative SportsCare Institute, New York, USA; 4 Physical Medicine and Rehabilitation, Interventional Orthopedics and Regenerative Medicine, Interventional Orthopedics of Atlanta, Atlanta, USA; 5 Department of Rehabilitation Medicine, Emory University School of Medicine, Atlanta, USA

**Keywords:** knee avascular necrosis, osteonecrosis, bone marrow concentrate

## Abstract

Osteonecrosis (ON) is a painful condition involving bony cell death with resultant architectural collapse. This report discusses the case of a 50-year-old Caucasian female who presented to an outpatient musculoskeletal clinic with severe chronic left knee pain. She had a history of ulcerative colitis and resultant chronic corticosteroid exposure with subsequent development of knee ON. She was treated with an intraosseous autologous bone marrow concentrate (BMC), demineralized bone matrix (DBM), and platelet-rich plasma (PRP) injection. At 11 months post-injection, she demonstrated a significant improvement in pain scores, mobility, activity, and decreased narcotic use. Intraosseous orthobiologic injection for the treatment of knee ON is a promising procedure with a reasonable safety profile that warrants further study as an alternative to surgical intervention.

## Introduction

Osteonecrosis (ON) is an often painful condition characterized by the architectural collapse of a bony structure due to cell death [1]. It is frequently debilitating and can lead to end-stage osteoarthritis [2]. After the hip, the knee is the second most common joint affected by ON [3,4]. Clinicians should consider ON as a cause of knee pain in the setting of corticosteroid use, trauma, high alcohol intake, and sickle cell disease [2,5]. ON of the knee can be classified into three subtypes: spontaneous primary ON of the knee (SONK), secondary ON, and post-arthroscopic ON. SONK, or primary ON of the knee, is the most common subtype and is predominantly associated with individuals over the age of 50 years [2]. The pathogenesis underlying SONK is attributed to subchondral insufficiency fractures in diseased and osteopenic bone, rather than from ischemia [2]. Secondary ON, also called avascular necrosis (AVN), is atraumatic and more prevalent in patients aged younger than 45 years. It is most commonly associated with corticosteroid exposure, alcohol consumption, sickle cell disease, and tobacco use [2,5]. Secondary ON occurs due to ischemia due to an interruption in vascular supply to the bony tissue. As the disease process progresses to advanced stages, bony destruction and subchondral collapse may occur [6-8]. Post-arthroscopic ON is the least common subtype and is reported to affect only 4% of patients post-knee arthroscopy, particularly following meniscectomy [2]. Patients most commonly present with acute knee pain associated with weight-bearing and occurring at night. This is often localized over the medial compartment and is not precipitated by trauma. While the prevalence rate of secondary knee ON is unknown, the overall prevalence of AVN with glucocorticoid use ranges from 3% to 38% [9].

MRI and radiographs are the mainstays of diagnosis, with MRI having the highest sensitivity for early detection. There should be a high index of suspicion for ON in bone and joint pain in the setting of long-term glucocorticoid use, and early MRI evaluation should be considered. Pathological features of AVN include heterogeneous MRI signals resulting from bone marrow necrosis after blood supply interruption [9]. Multiple radiographic staging systems exist for ON: the Modified Ficat and Arlet [5-8]; the Kashino, which was originally developed for SONK; and a version of Kashino modified by Aglietti [2,4,5,7,8]. The Ficat and Arlet staging, which is most commonly used for hip AVN, is based on radiographs and has been adapted in a modified form for application to the knee [4,5,7,8,9]. Stage I shows no evidence of ON. In stage II, there is visible perilesional sclerosis. Stage III shows early subchondral collapse with osteoarthritis formation, and stage IV demonstrates the progressive collapse of the condyle involved [7,8].

Currently, treatment options include nonoperative observation, analgesia, corticosteroid injections, and protected weight-bearing. Surgical interventions include joint-preserving surgery, total knee arthroplasty (TKA), unilateral knee arthroplasty (UKA), core decompression, and high tibial osteotomy [2]. Since the late 1990s, investigations of biologic materials, including autologous bone marrow concentrate (BMC) and platelet-rich plasma (PRP), have shown promise in treating a variety of musculoskeletal pathologies [10-15]. For bone marrow lesions and AVN in particular, the proposed therapeutic mechanism of action involves the repopulation of the subchondral microenvironment with healthier mesenchymal signaling cells (MSCs) transferred via concentrated bone marrow aspirated from a different anatomic location [14]. Over time, procedural techniques for minimally invasive percutaneous applications of these therapies have spawned the novel field of interventional orthopedics to serve as a bridge between traditional conservative care and surgery [16]. We present a case of secondary ON of the proximal tibia distal to the weight-bearing surface, as well as in the distal femur, which was successfully treated with a combination of intraosseous BMC, demineralized bone matrix (DBM), and PRP injection.

## Case presentation

A 50-year-old, 5'1" tall, 140-pound Caucasian female was seen at a specialty outpatient orthopedic practice with a chief complaint of severe left knee pain. She had a medical history significant for prolonged glucocorticoid exposure for the treatment of ulcerative colitis. She had undergone colectomy in 1994, following which daily corticosteroid administration had been replaced with intermittent, pulse-dosed glucocorticoids, as required. The onset of left knee pain had occurred in March of 2019 with a rapid increase in pain severity. She had developed difficulty in walking, necessitating the use of a cane for ambulation, and had started taking ibuprofen on a regular basis. Three months later, she had been evaluated by a rheumatologist and found to have bone infarcts of both the femur and tibia. This had led to the diagnosis of ON of the knee, presumably corticosteroid-induced due to exposure history. At that time, she had started alendronate and calcitonin. However, the pain had persisted and she had been prescribed oral morphine 15-30 mg every eight hours as needed by her primary care physician (PCP), following which she had been transitioned to a 25 mcg/hr fentanyl patch six months after the initial presentation due to worsening pain.

Despite additional analgesics, the pain had progressed with new-onset radiation into the distal tibia, ankle, and foot. This had prompted an evaluation with an orthopedic surgeon, who did not recommend surgical intervention. A second surgical opinion had been obtained with the recommendation to undergo intraarticular corticosteroid injections (CSI) for symptom management until the degree of progressive collapse necessitated TKA. A third surgical opinion had recommended against TKA since the location of the lesion in the tibia was too distal to the joint line and had instead considered transfemoral amputation as the best surgical option. A CSI had also been performed in September 2019 with no improvement. In October 2019, as her debilitating pain had continued, her PCP had increased her fentanyl patch dose to 37.5 mcg/hr, with oral morphine for breakthrough pain. At our evaluation in November 2019, her pain was 8/10 with ambulation and 5/10 with rest on the visual analog scale (VAS).

**Figure 1:**
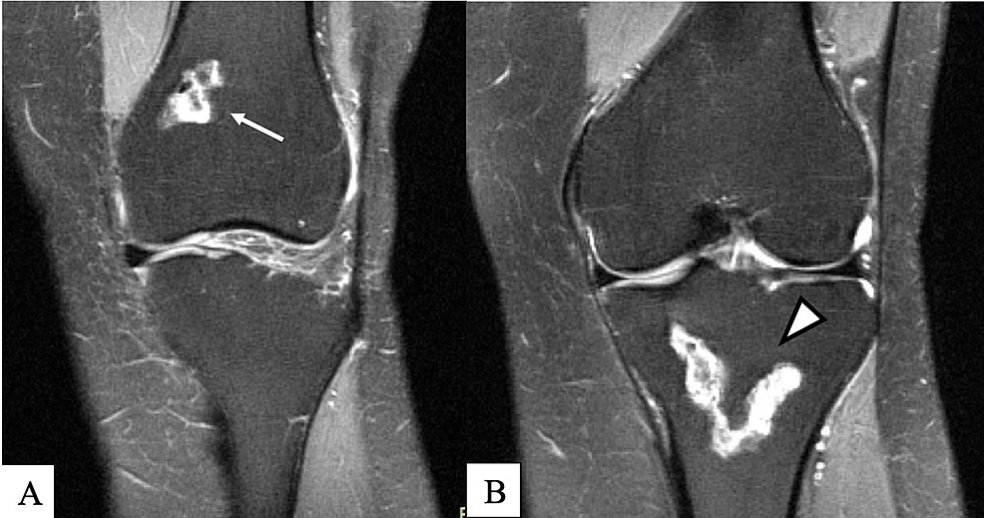
MRI dated June 2019 Proton density fat saturation without contrast; coronal view demonstrates bone infarct of the distal femoral metaphysis extending to the proximal aspect of the medial femoral condyle (arrow) (A) and bone infarct of the proximal tibial metaphysis with extension into the tibial plateau and periosteal reaction along the tibial metaphysis (arrowhead) (B) MRI: magnetic resonance imaging

**Figure 2:**
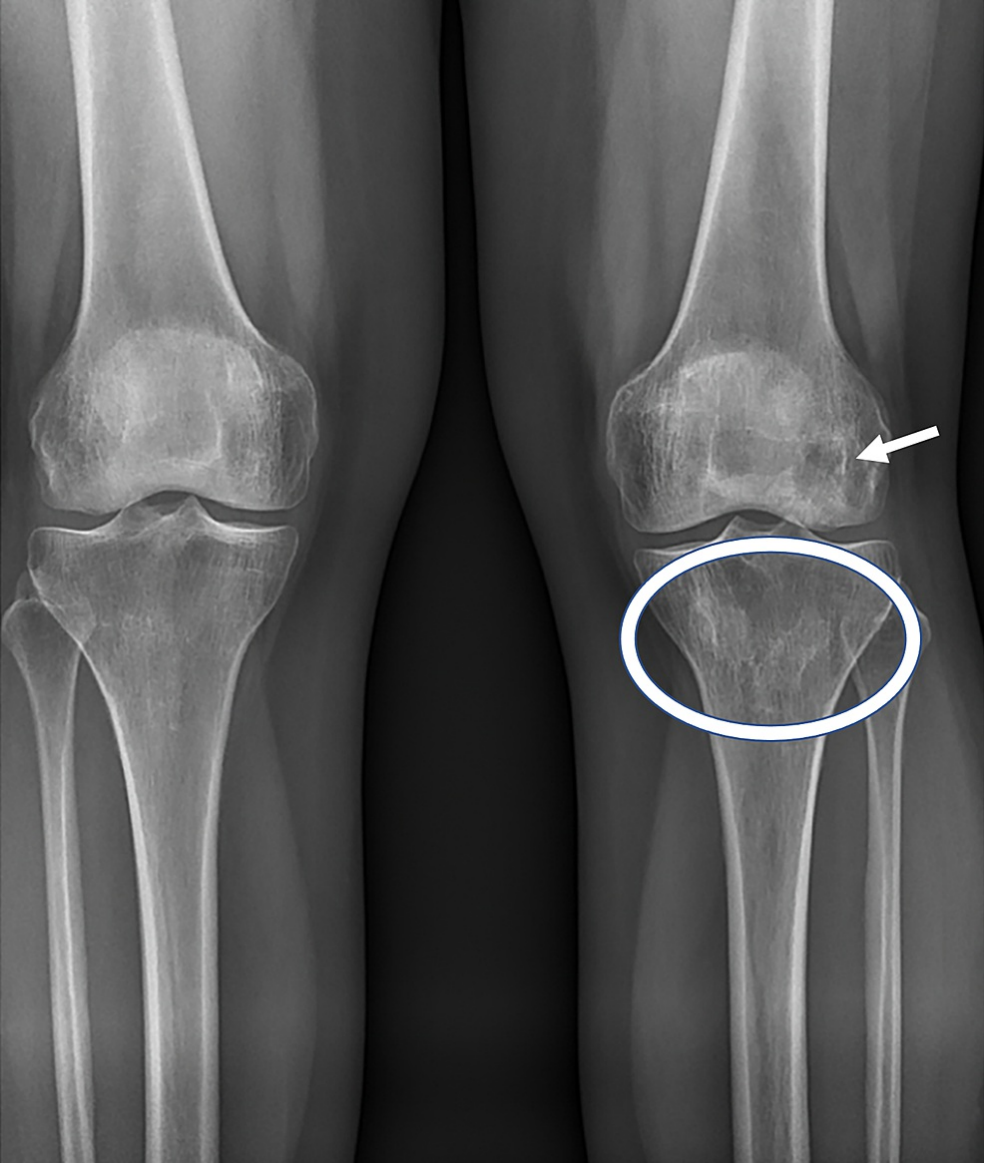
Anteroposterior radiographs of bilateral knees from September 16, 2019 The image demonstrates osteopenia and left distal femur (arrow) and proximal tibial (circle) areas of sclerosis, consistent with known bony infarcts

The patient arrived in a wheelchair for the clinical exam. Unassisted ambulation demonstrated an antalgic gait. She was tender to palpation over the left tibial tuberosity and had moderate laxity with a clear stopping point upon varus testing. She demonstrated 3/5 strength in left hip flexion, knee extension and flexion, and 4/5 strength in bilateral extensor hallucis longus muscles. No other significant findings were noted in the physical exam. MRI review from five months prior demonstrated medullary infarcts of the distal femoral metaphysis extending into the proximal aspect of the medial femoral condyle and of the proximal tibial metaphysis with extension into the tibial plateau (Figure 1). X-rays from three months prior to the visit were notable for osteopenia and Modified Ficat and Arlet stage II ON (Figure 2). Due to the failure of previous conservative management and the patient's desire to avoid amputation, an in-depth discussion was held about nonsurgical interventions. The patient elected to proceed with intraosseous treatment to the proximal tibia and distal femur with BMC, DBM, and leukocyte-poor 10x concentrated PRP in November 2019. Prior to undergoing bone marrow aspiration, corticosteroids and nonsteroidal anti-inflammatory drugs (NSAIDs) use were restricted for the patient for a minimum of six weeks and 14 days, respectively.

**Figure 3:**
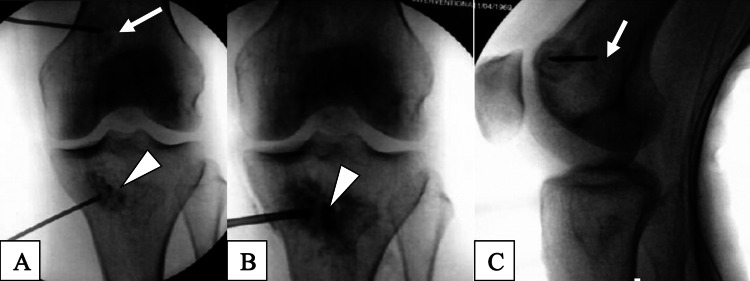
Fluoroscopy of the left knee on November 26, 2019 Anteroposterior (A & B) and lateral (C) views demonstrate intraosseous needles inserted at the bone infarct locations in the distal femoral metaphysis (arrows) and the proximal tibial metaphysis (arrowheads) each outlined by contrast dye

On the day of the procedure, bone marrow was harvested from the iliac crest under sterile conditions with ultrasound guidance. Following local anesthesia with 1% lidocaine and 0.25% ropivacaine, an 11-gauge bone marrow biopsy needle was utilized to obtain 45 cc of bone marrow aspirate (BMA) from four locations along the posterior superior iliac spine (PSIS) on each side of the pelvis, for a total of 90 cc. The BMA was hand-processed in a sterile ISO-7 cleanroom under ISO-5 laminar flow cabinets. The buffy coat was first isolated through centrifugation, producing 1-5 mL of BMC. This was transported sterilely to the procedure room for use. The PRP was prepared with 200x g centrifugation to separate the plasma and buffy coat from the red blood cells (RBC). The subsequent remaining supernatant was RBC- and leukocyte-poor. On the same day, with the patient under monitored anesthesia care and after full surgical prep, 15-gauge and 11-gauge 1.5-inch intraosseous needles were manually placed into the lesions in the medial femoral condyle and medial tibia plateau, respectively, under fluoroscopic guidance (Figure 3). The tibial lesion was injected with 7 cc of BMC, 1 cc of Arthrex AlloSync Demineralized Bone Matrix Putty (Arthrex, Naples, Fl), and 8 cc of autologous 10x leukocyte-poor PRP. The femoral lesion was injected with the same injectates, but with a volume of 3 cc of BMC (12 cc total volume), as it was a smaller lesion. The BMC had a total nucleated cell count of 3.5 billion and viability of 89%, as determined by a Bio-Rad TC-20 automated cell counter (Bio-Rad Laboratories, Inc., Hercules, CA). After the procedure, the patient was advised to continue weight-bearing as could be tolerated while wearing a knee unloader brace until her first follow-up at six months post-procedure.

**Figure 4:**
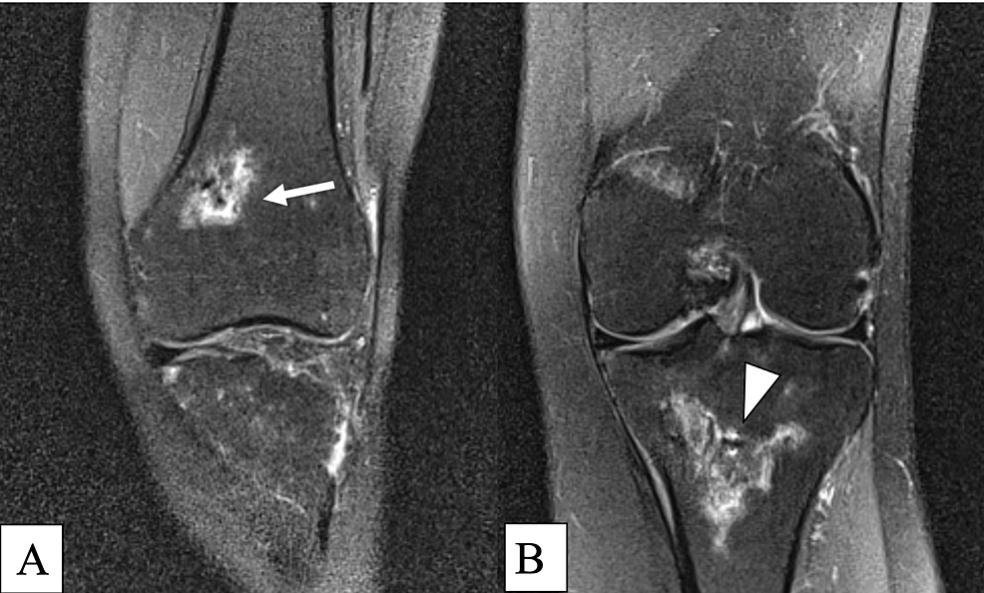
1.5 Tesla MRI dated August 2020 Proton density fat saturation without contrast; coronal view demonstrates the unchanged size of bone infarcts but shows an interval decrease in surrounding bone marrow edema in both the distal femoral metaphysis (arrow) (A) and the proximal tibial metaphysis (arrowhead) (B) MRI: magnetic resonance imaging

The patient tolerated the procedure well and continued with minimal activity following the injection. At two months post-procedure, she initiated physical therapy with a focus on aquatic therapy but was unable to tolerate it due to severe pain. However, at the three-month mark, she began to report a gradual improvement in pain and function. Nine months following the procedure in August 2020, she was able to participate in aquatic therapy without pain, with progression to land-based physical therapy after three sessions. Her VAS was noted to be 0/10 pain with rest and household ambulation at her nine-month follow-up. She no longer required a wheelchair, unloader brace, or any assistive devices other than a knee sleeve. Her narcotic load had also decreased. Though still requiring the fentanyl patch at 37.5 mcg/hr, she no longer needed breakthrough oral morphine for pain control. Her most recent MRI obtained at nine months post-procedure showed an unchanged size of the bone infarcts in the distal femur and proximal tibia; however, there was a noted interval decrease in the extensive surrounding bone edema (Figure 4). At 15 months post-procedure, she was no longer taking any opioids for breakthrough pain and was slowly titrating down the fentanyl patch. Her pain was still a 0-1/10 at its worst, intermittent in nature, and most notable with increased activity. She had resumed all of her pre-morbid activities without any assistive devices, including ambulation.

## Discussion

ON has numerous etiologies with multiple contributing factors. Depending on the type of ON, the pathologic process can be related to ischemia, subchondral collapse, or a combination of the two. The current approach to the treatment of ON of the knee varies greatly. Secondary ON often progresses to end-stage disease, with bone ischemia leading to subchondral fracture, collapse, and subsequent joint failure. This process often results in debilitating pain requiring the need for TKA or other surgical intervention [4]. Mont et al. have reported a nonoperative treatment success rate of only 20% in patients with secondary knee ON [5]. Therefore, surgical intervention is typically recommended, specifically core decompression for patients without and TKA for patients with subchondral collapse. However, surgery carries with it an increased risk of postoperative complications, as well as others related to anesthesia. Indeed, TKA is effective in treating 74% of knee ON cases, with a 20% revision rate [5]. Therefore, clinicians have investigated alternative interval treatments.

In 2018, Hernigou et al. published a prospective randomized controlled trial involving 30 patients (60 knees) with bilateral steroid-induced stage IV knee ON. Each patient underwent treatment with TKA on one side and subchondral BMC delivered via percutaneous injection on the contralateral side. At an average follow-up of 12 years post-procedure, Knee Scores were similar among groups (80.3 points in TKA versus 78.3 in BMC); however, there were greater patient satisfaction and fewer complications reported in the subchondral BMC sides. Furthermore, on follow-up imaging, improvements in bone marrow lesions were found in the BMC group. This study included a comparative analysis of MSCs per mL of bone marrow aspirated from the diseased areas of the femurs and tibias to that of the iliac crests, from which aspirate was harvested to produce BMC. The results showed a greater than 10-fold decrease in MSCs per mL in the diseased bone compared with the iliac crest samples [14]. Therefore, the proposed mechanism of action for therapeutic efficacy is the use of bone marrow harvested and concentrated from a healthier location as a method of repopulating MSCs in the diseased ON lesions. In addition, one case report has discussed an 18-year-old female with steroid-induced bilateral knee ON (Ficat IV) treated with five separate subchondral injections using BMC with the resolution of pain in three months, improvement of bone marrow edema, and bone maturation on repeat MRI at 12 months, and significant improvement in Knee Injury and Osteoarthritis Outcome Score (KOOS) and Western Ontario and McMaster Universities Osteoarthritis Index (WOMAC) scores at 24 months [15]. Hernigou et al. also demonstrated the ability of intraosseous implantation of BMCs to significantly postpone arthroplasty in patients with knee osteoarthritis [10].

There are many factors to consider with regard to the application of orthobiologic therapies. Some contraindications include active malignancy, a history of lymphoma or leukemia, bleeding disorders, and active infection. The benefit of attempting a less invasive approach is the potential for decreased morbidity related to surgery. However, in the event that an orthobiolgic injection is not effective, the patient risks possible further progression or advancement of subchondral collapse with delayed surgical intervention while awaiting the procedure to demonstrate efficacy. In our patient, the surgical option was above-knee amputation due to the proximal location of the femoral lesion in the metaphysis. Instead, we were able to successfully treat her pain and dysfunction with follow-up spanning 15 months post-procedure with an intraosseous injection of BMC into her avascular lesions. This case report adds to the growing body of literature in support of this novel method of treating knee ON with percutaneous BMC implantation as an alternative to surgical intervention. In this patient, such a treatment prevented the need for transfemoral amputation and enabled a return to premorbid levels of activity.

## Conclusions

In this case, the intraosseous injection of BMC, DBM, and PRP demonstrated great efficacy and a good safety profile for the treatment of severely debilitating knee ON. Clinical improvement and some reversal of medullary necrosis were observed post-treatment. In pre-collapse patients or patients with medullary ON, interventional orthobiologics may be a reasonable management option. Larger clinical trials are needed to further study the long-term efficacy of such treatments as potential alternatives or adjuncts to traditional surgical management for patients for whom conservative medical management has failed.
